# Cerium Oxide Nanoparticles Protect Cortical Astrocytes from Oxygen–Glucose Deprivation through Activation of the Ca^2+^ Signaling System

**DOI:** 10.3390/ijms241814305

**Published:** 2023-09-19

**Authors:** Elena G. Varlamova, Alexey S. Baryshev, Sergey V. Gudkov, Valentina A. Babenko, Egor Y. Plotnikov, Egor A. Turovsky

**Affiliations:** 1 Institute of Cell Biophysics of the Russian Academy of Sciences, Federal Research Center “Pushchino Scientific Center for Biological Research of the Russian Academy of Sciences”, 142290 Pushchino, Russia; 2 Prokhorov General Physics Institute of the Russian Academy of Sciences, 38 Vavilove St., 119991 Moscow, Russia; 3 A.N. Belozersky Institute of Physico-Chemical Biology, Lomonosov Moscow State University, 119992 Moscow, Russia; 4 V.I. Kulakov National Medical Research Center of Obstetrics, Gynecology and Perinatology, 117997 Moscow, Russia

**Keywords:** ROS production, intracellular calcium ions, signaling, selenoproteins, cerium oxide nanoparticles, apoptosis, necrosis, oxygen–glucose deprivation, cytoprotection, cortical astrocytes, gene expression

## Abstract

Most of the works aimed at studying the cytoprotective properties of nanocerium are usually focused on the mechanisms of regulation of the redox status in cells while the complex effects of nanocerium on calcium homeostasis, the expression of pro-apoptotic and protective proteins are generally overlooked. There is a problem of a strong dependence of the effects of cerium oxide nanoparticles on their size, method of preparation and origin, which significantly limits their use in medicine. In this study, using the methods of molecular biology, immunocytochemistry, fluorescence microscopy and inhibitory analysis, the cytoprotective effect of cerium oxide nanoparticles obtained by laser ablation on cultured astrocytes of the cerebral cortex under oxygen–glucose deprivation (OGD) and reoxygenation (ischemia-like conditions) are shown. The concentration effects of cerium oxide nanoparticles on ROS production by astrocytes in an acute experiment and the effects of cell pre-incubation with nanocerium on ROS production under OGD conditions were studied. The dose dependence for nanocerium protection of cortical astrocytes from a global increase in calcium ions during oxygen–glucose deprivation and cell death were demonstrated. The concentration range of cerium oxide nanoparticles at which they have a pro-oxidant effect on cells has been identified. The effect of nanocerium concentrations on astrocyte preconditioning, accompanied by increased expression of protective proteins and limited ROS production induced by oxygen–glucose deprivation, has been investigated. In particular, a correlation was found between an increase in the concentration of cytosolic calcium under the action of nanocerium and the suppression of cell death. As a result, the positive and negative effects of nanocerium under oxygen–glucose deprivation and reoxygenation in astrocytes were revealed at the molecular level. Nanocerium was found to act as a “double-edged sword” and to have a strictly defined concentration therapeutic “window”.

## 1. Introduction

Cerium (Ce, atomic number 58) is a soft, ductile silver-white metal, the second element in the lanthanide series, and although it often exhibits a (+3) oxidation state, it also has a stable (+4) state. In recent years, scientists working on miniaturized delivery systems have introduced drug delivery systems and vaccines based on nanoparticles [1]. The use of nanomaterials has led to great advances in the diagnosis and treatment of numerous pathologies. Nanotechnology is the branch of science which deals with particles in the range of 1–100 nm. These particles are called nanoparticles, and they exhibit unique electronic, optical, magnetic, and mechanical properties, which make them different from the bulk material [2]. The shape of nanoparticles can influence biological effects as well as their size and surface charge [3]. In our experiments, we used spherical CeNPs. The shape effect of nanoparticles is mainly determined by different membrane-bending energies during endocytosis. A major advantage of the spherical shape of nanoparticles for applications in biomedicine is the minimal membrane-bending energy barrier than the non-spherical counterparts [4,5]. It has been shown that the antioxidant and anti-inflammatory activities in mild traumatic brain injury in vivo did not differ significantly depending on the nanoshape, although the mRNA expressions of SOD1 and SOD2, as well as COX-2 tended to decrease in ceria nanorods than in ceria nanospheres [5]. In recent years, a cerium oxide nanoparticle (CeNP) has gained popularity in biomedicine. An important feature of cerium oxide nanoparticles is their ability to exhibit pro-oxidant and antioxidant properties depending on the conditions. It has been established that nanocerium has an antioxidant effect at physiological pH and pro-oxidant effects at acidic pH [6]. It is generally accepted that cancer cells function at an acidic intracellular pH, and that CeNPs could have a targeted prooxidant effect on cancer cells [7]. The radioprotective properties of nanocerium were demonstrated using human colon cells (CRL 1541 cell line). Pretreatment of these cells with various concentrations of nanoparticles for 24 h protected the cells from radiation through the suppression of oxidative stress and increased expression of superoxide dismutase 2 [8].

At the same time, nanocerium has a pronounced antitumor effect. Cancer cells have a more acidic environment compared to healthy cells, which is due to an increased rate of glycolysis and lactic acid production [9]. In the acidic environment (low pH), the antioxidant (cytoprotective) activity of nanoceria is lost, and it acts as a prooxidant which causes oxidative stress by producing ROS and thus induces cell death/apoptosis [10]. It has been shown that nanocerium at a concentration of 5 mg/mL has cytotoxic effects on the prostate cancer cell line (PC3), but has no effect on normal mouse fibroblasts of the L-929 line [11]. The toxicity of nanoceria depends upon various factors, such as particle size, preparation method, cell type, dose/concentration, exposure time, and exposure route [12]. That is, depending on the production technology, diameter, and target, nanocerium can have both cytoprotective and cytotoxic effects.

Ischemic stroke is the second leading cause of death in the world. During ischemia and reoxygenation, there is an increased generation of ROS, such as the superoxide anion, hydrogen peroxide, and hydroxyl radical. The accumulation of excess intracellular ROS leads to oxidative stress and activation of cell death pathways. As a result, the destruction of neuronal networks and neurovascular units occurs, which leads to foci of brain damage [13,14,15]. CeNPs are known to have neuroprotective and cardioprotective properties, which are realized through their antioxidant or redox modulating action [16,17]. The antioxidant property of nanocerium is primarily explained by its ability to bind oxygen and shifting between the Ce^3+^ (reduced) and Ce^4+^ (oxidized) forms at the particle surface [18]. Cerium ions has been shown to exist mainly in the Ce^4+^ valence state; however, a decrease in the particle size leads to the appearance of oxygen vacancies on the particle surface, which makes possible the coexistence of Ce^3+^ [19]. Interestingly, the catalytic properties of cerium oxide nanoparticles can be further improved by using ultra-small nanoparticles with a size of less than 4 nm [20]. It is known that spherical CeNPs reduced neuronal death and calcium dysregulation by preserving the antioxidant system in models of traumatic brain injury [21]. After pre-incubation of C17.2 cells with nanocerium and subsequent treatment with 2,3-dimethoxy-1,4-naphthoquinone (10 µM), a significant suppression of ROS production and cell death was found. The antioxidant effect of CeNPs began to be recorded at a concentration of 20 µg/mL. However, this effect was transient and ultimately did not suppress cell death. It was also found that a decrease in basic ROS production under the effect of nanocerium led to the reduction in both the neuronal and neuroglial differentiation of the C17.2 neural stem cells [22]. On the other hand, it was shown in PC12 cells that nanocerium enhances dopamine secretion and neurite outgrowth, which is a sign of enhanced cell differentiation [23]. Thus, knowledge about the effects of nanocerium on brain cells remains controversial and was obtained mainly on progenitor cell lines capable of differentiating into neurons. Nevertheless, it can be said that nanocerium acts similar to a double-edged sword on “neuron-like” cells, when its antioxidant properties can cause disruption of cell differentiation, since ROS are extremely important for neuronal differentiation [24]. As for astrocytes, the most numerous types of brain cells, there are practically no data on the effect of nanocerium on their physiology, especially under conditions of ischemia/reoxygenation.

Thus, the search for new cytoprotectors capable of penetrating the blood–brain barrier and protecting astrocytes from ischemia and reoxygenation is an extremely urgent task in modern biomedicine. The properties of cerium in the nanoform can meet the desired requirements, and the study of the mechanisms of the protective effect of nanocerium will contribute to the development of new approaches for the prevention and treatment of stroke. In this study, we used a whole arsenal of methods and approaches that were previously used to study the cytoprotective effect of selenium nanoparticles [25,26,27,28,29]. The approaches we have used have been clearly developed over many years of research and allow us to perform a comprehensive study of the mechanisms for nanoparticles of various origins. Thus, nanocerium can be both a source of ROS and its scavenger. At the same time, the functioning of neuroglial networks, both under normal conditions and during ischemia, is subject to the control of redox systems, while ROS act both as signaling molecules and as sources of oxidative stress. The known mechanisms of the neuroprotective action of nanocerium are based on its physical properties. In addition, there is limited data on its role in the regulation of the expression of genes encoding protective proteins, on the regulation of the dynamics of cytosolic calcium, and the relationship between these processes and cell death. This comprehensive study is aimed at elucidating the mechanisms and dose dependencies of the protective effect of nanocerium under oxygen–glucose deprivation.

## 2. Results

### 2.1. The Effects of Cerium Oxide Nanoparticles on ROS Production by Astrocytes in the Cerebral Cortex

**Figure 1:**
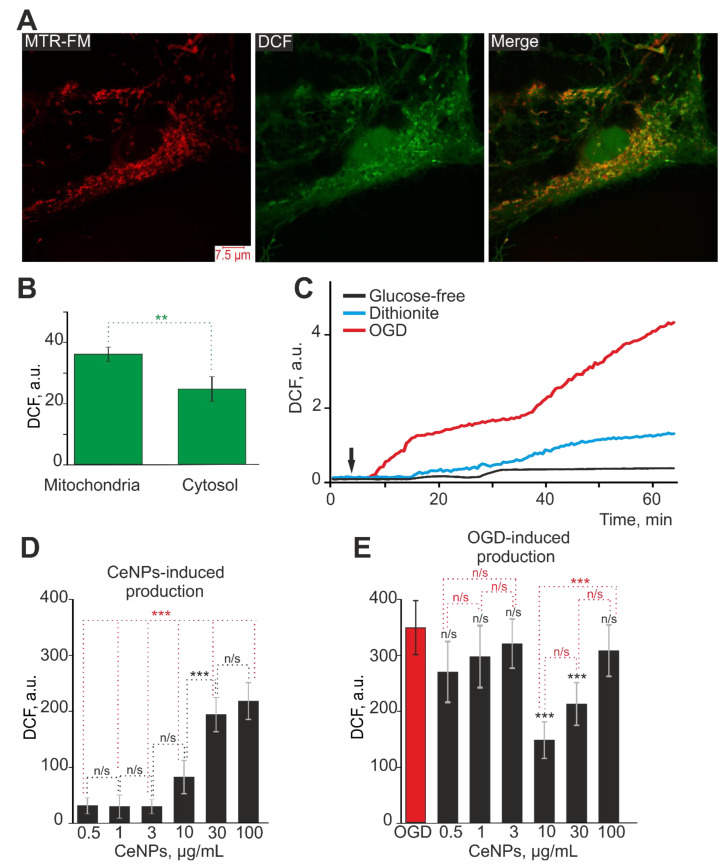
Dose-dependent effect of CeNPs on cortical astrocytes ROS production in acute experiments (**D**) and after 24 h pre-incubation with CeNPs on OGD-induced ROS production (**E**). (**A**)—Simultaneous staining of cortical astrocytes with a DCF probe (to measure ROS production) and a mitochondrial probe (MitoTracker Red FM, MTR-FM). (**B**)—Localization of DCF in the mitochondria and cytosol of cortical astrocytes, expressed in units of DCF fluorescence. Statistical analysis was performed with paired *t*-test (** *p* < 0.01). (**C**)—ROS production in cortical astrocytes in response to glucose-free media (black curve), application of the oxygen scavenger sodium dithionite to HBSS with glucose (blue curve) and oxygen–glucose deprivation (OGD, lasting 55–58 min) modeled by application of sodium dithionite in glucose-free media (red curve). The curves of ROS production averaged over several dozens of cells, imaged with a fluorescece microscope are presented. (**D**)—Effect of various concentrations of CeNPs on astrocytic ROS production. (**E**)—Effect of 24 h pre-incubation of cortical astrocytes with different concentration of CeNPs on ROS production induced by OGD (glucose-free + sodium dithionite lasting 55–58 min). For panels (**D**,**E**) data obtained with an automated multiplate reader (Spark™ 10 M multimode microplate reader, Tecan Trading AG, Zurich, Switzerland) are presented. Data are shown as the mean fluorescence intensity, arb.units ± S.E.M. Statistical analysis was performed with paired *t*-test. Statistical significance was assessed using *t*-test. For panel (**D**), the comparison between experimental groups is in black, and in red is the statistical comparison between CeNPs concentrations of 0.5–10 µg/mL and 30–100 µg/mL. For panel (**E**), the differences between the OGD group and CeNPs are marked in black, while the differences between the experimental groups of CeNPs are marked in red. *** *p* < 0.001, n/s—no significant differences.

Double simultaneous staining of astrocytes in the cerebral cortex with the ROS-sensitive probe H2DCF-DA (DCF) and the mitochondrial marker Mito Tracker Red (MTR-FM) revealed the localization of the DCF probe both in the cytoplasm of astrocytes and its co-localization in mitochondria (Figure 1A). ImageJ was used to distinguish mitochondria stained with MitoTracker Red and to measure DCF fluorescence in these areas, which reflects the ROS level in mitochondria. In contrast, the areas without MitoTracker Red fluorescence reflect the ROS level in the cytosol (Figure 1C). Thus, using H2DCF-DA, it seems possible to detect the total (cytosolic and mitochondrial) ROS production in astrocytes under various exposures. To record the dynamics of total ROS production by astrocytes, a series of images were acquired with a fluorescence recording interval of 1 frame per 30 s, which made it possible to avoid photodynamic ROS production and probe destruction.

Oxygen–glucose deprivation (OGD) lasting 55–58 min was performed by replacing HBSS (Hanks’ balanced salt solution) containing 10 mM HEPES with HBSS without glucose supplemented with the oxygen scavenger, sodium dithionite (Figure 1C, red curve). It was found that after a short lag period, OGD caused a biphasic increase in ROS production in astrocytes (Figure 1C—red curve), while glucose-free media (Figure 1C—black curve) or application of sodium dithionite to the medium with glucose (Figure 1C—blue curve) caused a biphasic increase in ROS production with a less pronounced first phase signal and significantly smaller increase in ROS production.

Since it is known that nanocerium can have both prooxidant and antioxidant effects [7,16,17], it was reasonable to study the dose effects of CeNPs in an acute experiment and the effect of 24 h pre-incubation of astrocytes with CeNPs on OGD-induced ROS production. Application of various concentrations of CeNPs to astrocytes in an acute experiment (duration 55 min) showed that the application of 10–100 µg/mL CeNPs caused a dose-dependent increase in ROS production, while CeNPs concentrations of 0.5–3 µg/mL did not affect this process (Figure 1D). At the same time, 24 h pre-incubation of astrocytes with CeNPs in the concentration range of 0.5–3 µg/mL and at high concentrations (100 µg/mL) had no significant effect on OGD-induced ROS production (Figure 1E). However, 24 h pre-incubation of astrocytes with 10 or 30 µg/mL led to a significant suppression of the OGD-induced increase in ROS production (Figure 1E).

Thus, CeNPs at concentrations of 10–100 µg/mL can themselves lead to the generation of ROS by astrocytes in an acute experiment, but, on the other hand, pre-incubation of astrocytes with 10 or 30 µg/mL CeNPs led to inhibition of OGD-induced ROS production. This effect can be explained by the preconditioning effect of CeNPs in the concentration range of 10–30 µg/mL. After 24 h of exposure to nanoparticles, astrocytes have been adapted to the upcoming OGD-induced oxidative stress.

### 2.2. Activation of the Ca^2+^ Signaling System in Astrocytes of the Cerebral Cortex in Response to Cerium Oxide Nanoparticles. The Role of Nanocerium in Suppressing the Global Increase in Cytosolic Calcium in OGD

**Figure 2:**
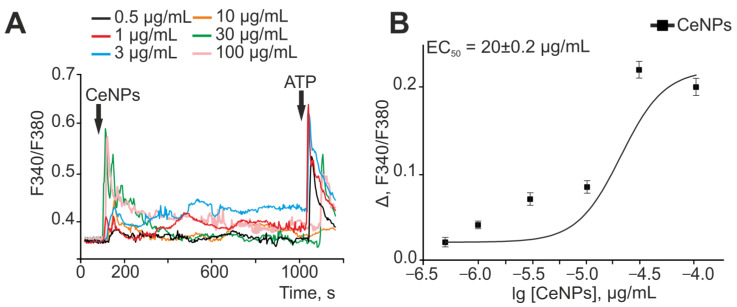
Application of various concentrations of CeNPs causes the generation of Ca^2+^ signals in astrocytes of the cerebral cortex. (**A**)—Averaged Ca^2+^ signals in astrocytes of the cerebral cortex upon application of increasing concentrations of CeNPs and 10 μM ATP. (**B**)—Dependence of the amplitude of Ca^2+^ responses of astrocytes on the CeNPs application and its approximation by a sigmoid function. For panel (**B**), the amplitude of Ca^2+^ responses were calculated as Δ = Fmax − Fmin, where Fmax is the maximal cellular Ca^2+^ signal to CeNPs application; Fmin is the Fura-2 fluorescence level before application of CeNPs (basal level).

It is known that ROS production in cells is associated with and regulated by an increase in the concentration of cytosolic Ca^2+^ ([Ca^2+^]_i_) [30,31]. Application of increasing concentrations of CeNPs to cortical astrocytes caused the generation of Ca^2+^ signals even after application of 0.5 µg/mL nanoparticles. An increase in the concentration of CeNPs resulted in a dose-dependent increase in the amplitudes of Ca^2+^ signals (Figure 2), which had the form of a transient increase in [Ca^2+^]_i_ and usually with the establishment of a new steady state for [Ca^2+^]_i_ (Figure 2A). Approximation of the amplitudes of the Ca^2+^ signals from astrocytes to the addition of various concentrations of CeNPs using the sigmoid function showed that the EC50 for these nanoparticles was 20 ± 0.2 µg/mL (Figure 2B). Staining of astrocyte cell cultures with fluorescent probe propidium iodide after the acute experiments presented did not reveal CeNPs-induced cell death (Supplementary Figure S1), since the culture always contains single dead cells, and after the application of CeNPs there was no massive increase in PI-positive astrocytes.

When CeNPs were applied to cortical neuroglial cultures, only astrocytes responded with an increase in [Ca^2+^]_i_ (Supplementary Figure S2A), and no Ca^2+^ signals were observed in neurons (Supplementary Figure S2B).

**Figure 3:**
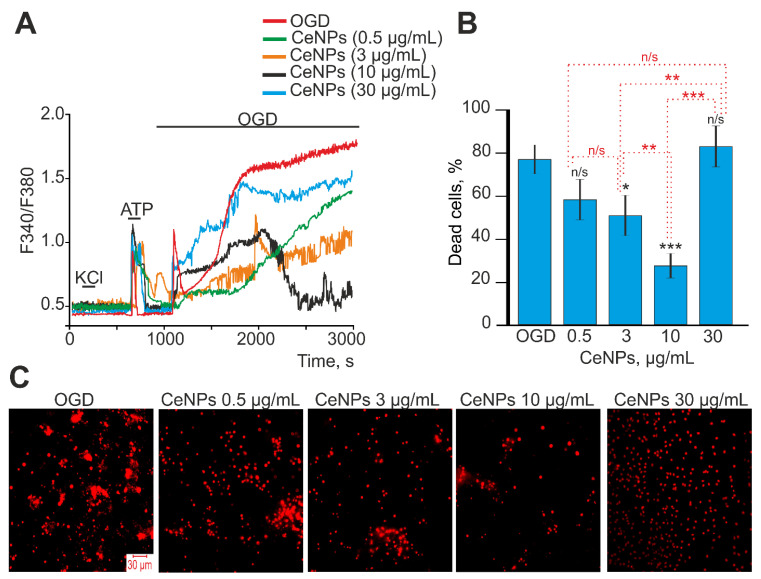
Effect of pre-incubation of cortical astrocytes with different concentrations of CeNPs on Ca^2+^ signal generation and survival after OGD. (**A**)—Ca^2+^ signals of astrocytes during OGD (40 min) and OGD after 24 h of pre-incubation with different concentrations of CeNPs. Short-term applications of 35 mM KCl and 10 µm ATP were used to distinguish neurons and astrocytes, respectively. Ca^2+^ signals from astrocytes averaged over several dozens of cells are presented. The experiments were performed in three replicates on three different cell cultures. (**B**)—Effect of 24 h pre-incubation with CeNPs on the cell viability after 40 min OGD. Statistical significance was assessed using the *t*-test. Black asterisks indicate the differences between the experimental groups compared with the OGD group. Differences between experimental groups are indicated with red asterisks. *** *p* < 0.001, ** *p* < 0.01, and * *p* < 0.05. n/s—no significant differences. (**C**)—Images of cortical astrocytes loaded with propidium iodide (PI) after 40-min OGD. OGD conditions induced by omission of O_2_ in glucose-free media with argon bubbling. Cell membrane disruption after 40 min of OGD and the appearance of PI fluorescence indicates necrotic cell death. The red dots represent the PI-stained nuclei of the necrotic cells.

Oxygen–glucose deprivation for 40 min caused biphasic Ca^2+^ signals in cortical astrocytes (Figure 3A—red curve), which led to necrotic death of up to 80% of cells in the microscope field (Figure 3B,C—OGD). The first phase of the OGD-induced increase in [Ca^2+^]_i_ is reversible and is unlikely to be the cause of the rapid death of astrocytes. The second phase of the OGD-induced increase in [Ca^2+^]_i_ is a global irreversible increase in [Ca^2+^]_i_, which can lead to necrosis. Pre-incubation of astrocytes for 24 h with 0.5 or 30 µg/mL CeNPs did not suppress both the transient and the global increase in [Ca^2+^]_i_ in astrocytes during OGD (Figure 3A, green and blue curves, respectively). OGD-induced death of astrocytes did not change significantly after pre-incubation with these concentrations of CeNPs (Figure 3B,C). However, pre-incubation of astrocytes with 3 µg/mL (Figure 3A, orange curve) or 10 µg/mL CeNPs resulted in suppression of the OGD-induced global increase in [Ca^2+^]_i_. In addition, pre-incubation of astrocytes with 10 μg/mL CeNPs resulted in a decrease in the [Ca^2+^]_i_ level after OGD to a resting level (Figure 3A, black curve), which may be the result of the activity of the cellular Ca^2+^ transporting systems. OGD-induced cell death after 24 h pre-incubation of astrocytes with 3 µg/mL CeNPs was up to 60% of cells, and after 10 µg/mL CeNPs, death decreased to 34% of cells (Figure 3B,C).

Thus, CeNPs are able to induce the generation of Ca^2+^ signals in astrocytes in an acute experiment in a dose-dependent manner with an EC50 of 20 ± 0.2 µg/mL. Pre-incubation of astrocytes with 3–10 µg/mL CeNPs resulted in a significant inhibition of the global increase in [Ca^2+^]_i_ during OGD and a decrease in the number of necrotic cells. At the same time, lower (0.5 µg/mL) or higher (30 µg/mL) concentrations of CeNPs did not affect the OGD-induced Ca^2+^ dynamics and cell death.

### 2.3. The Effects of CeNPs on Basal and Ischemia/Reoxygenation-Induced Expression of Genes Encoding Protein Regulators of Cell Death and Redox Status

**Figure 4:**
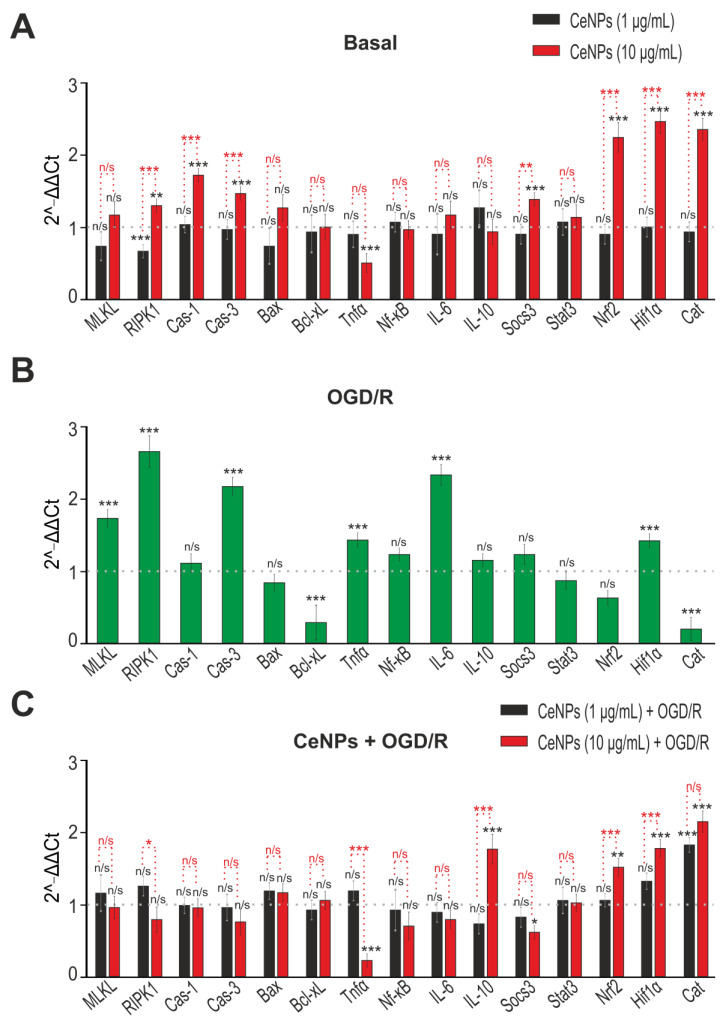
The effect of 24 h pre-incubation of cortical astrocytes with 1 or 10 μg/mL CeNPs on basal (**A**) and OGD/R-induced (**C**) gene expression. (**B**)—OGD/R-induced gene expression. Gene expression in intact cells is marked with a dashed line in panel (**A**,**B**). Gene expression in OGD/R cells without treatment is marked with a dashed line in panel (**C**). Statistical significance was assessed using the ordinary two-way ANOVA test with Geisser–Greenhouse correction. Comparison of experimental groups vs. control: n/s—no significant differences (*p* > 0.05), * *p* < 0.05, ** *p* < 0.01, and *** *p* < 0.001, *n* = 3. Comparison of experimental groups with each other is indicated in red.

Pre-incubation of cortical astrocytes with 1 µg/mL CeNPs for 24 h did not cause changes in basal expression of the genes studied, except for RIPK1 which was reduced by 25% (Figure 4A, black columns), whereas pre-incubation with 10 µg/mL CeNPs resulted in a two-fold increase in the expression of the Nrf2, Hif1α, and Cat genes, Socs3 increased by 44% and Tnfα decreased by 48% (Figure 4A, red columns). There was an increase in the expression of RIPK1 (42%), Cas-1 (73%) and Cas-3 (51%) after 24 h pre-incubation with 10 µg/mL CeNPs (Figure 4A, red columns).

After a 2 h OGD followed by a 24 h reoxygenation, there was a change in the expression of a number of genes encoding proteins involved in cell death (Figure 4B). There was a 1.5–2.8-fold increase in the expression of the MLKL, RIPK1, Cas-3, Tnfα, and IL-6 genes encoding proteins involved in cell death and inflammation. After OGD/R, there was an increase in the expression of the Hif1α gene, which is one of the protective proteins during ischemia. However, the expression of the anti-apoptotic and antioxidant genes Bcl-xL and Cat decreased after OGD/R (Figure 4B).

Pre-incubation with CeNPs at a concentration of 1 µg/mL caused only an increase in Cat mRNA expression (84%) after OGD/R (Figure 4C, black bars). Pre-incubation of astrocytes with 10 µg/mL CeNPs led to an increase in mRNA expression of the Nrf2 (67%), Hif1α (84%), Cat (two-fold), and IL-10 (87%) genes (Figure 4C, red columns).

**Figure 5:**
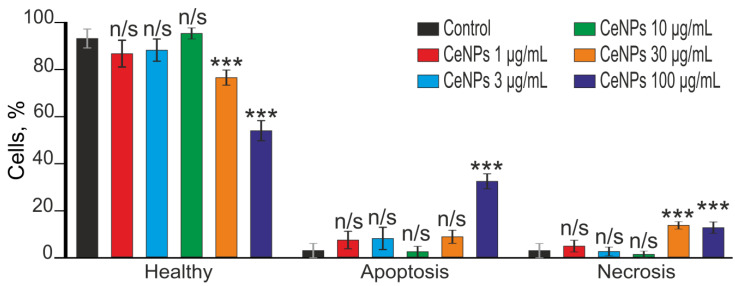
Effect of 24 h pre-incubation of cortical astrocytes with 1, 3, 10, 30 and 100 μg/mL CeNPs on the induction of necrosis and apoptosis. Cells staining using the Apoptosis/Necrosis Detection Kit assay (Supplementary Figure S3). Each value is the mean ± SE (*n* ≥ 3). Comparison of experimental groups with control: n/s—data not significant (*p* > 0.05), *** *p* < 0.001.

Staining of the astrocyte culture using the Apoptosis/Necrosis Detection Kit (Supplementary Figure S3) showed that without CeNPs (Control) exposure 94% of astrocytes were viable (Healthy), and apoptosis and necrosis were detected in 3% of cells (Figure 5). Pre-incubation of astrocytes with various CeNPs (1–100 μg/mL) concentrations for 24 h showed that 1, 3 and 10 μg/mL CeNPs concentrations was no induction of cell death, whereas after pre-incubation of astrocytes with 30 μg/mL or 100 μg/mL CeNPs, induction of apoptosis was observed in 8.9% and 32.7% of cells, and necrosis was registered in 13.9% and 12.9% of astrocytes (Figure 5; Supplementary Figure S3), respectively.

**Figure 6:**
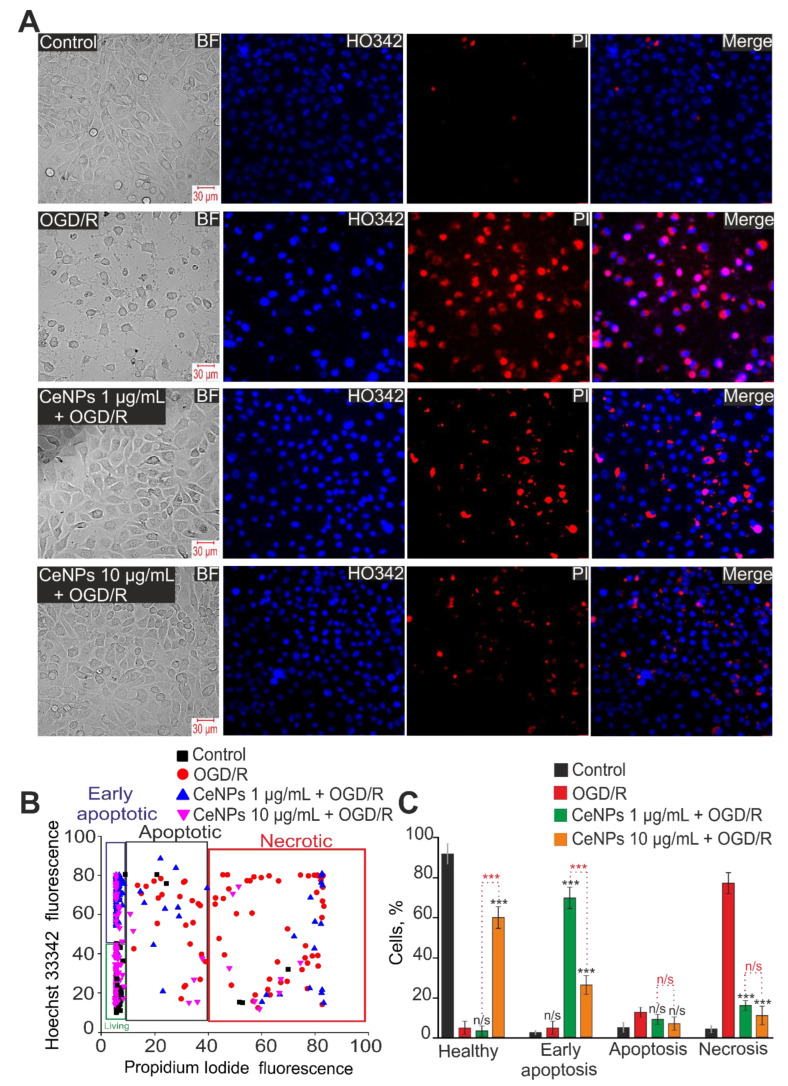
The effect of 24 h pre-incubation of cortical astrocytes with 1 or 10 μg/mL of CeNPs on the OGD/R-induced cell death. (**A**)—Double staining of cells with Hoechst 33342 (HO342), propidium iodide (PI), merge HO342 with PI and bright-field microscopy (BF). Control—cells without OGD/R. OGD/R—induction of OGD (2 h) and reoxygenation (24 h) without pretreatment with CeNPs. (**B**)—Cytogram demonstrating the viability of cortical astrocytes in Control (without OGD/R), after OGD/R (2 h and 24 h reoxygenation) and after 24 h pre-incubation with 1 or 10 μg/mL CeNPs and OGD/R. *X*-axis—the intensity of PI fluorescence; *Y*-axis—the intensity of Hoechst 33342 fluorescence. Cells were stained with the probes 24 h after the OGD/R. (**C**)—Effect of 24 h pre-incubation with 1 or 10 μg/mL CeNPs on the induction of necrosis and apoptosis. *n* of cell cultures = 3; *n* of cover slips with cells for each sample = 5. Statistical significance was assessed using the *t*-test. Comparison of experimental groups vs. OGD/R: n/s—no significant differences (*p* > 0.05), *** *p* < 0.001. Comparison of experimental groups with each other is indicated in red.

In the control group without OGD/R or CeNPs, approximately 92% of astrocytes remained viable, necrosis (by the presence of PI fluorescence) (Figure 6A,B—Control) was detected in 3% of cells, and early and late stages of apoptosis were detected in 1% and 4% astrocytes, respectively (Figure 6C). Viability assay for the astrocytes after OGD/R showed that approximately 3% of cells remained viable, necrosis was detected in 79% of cells, and early and late apoptoses in 4% and 14%, respectively (Figure 6—OGD/R). Pre-incubation of astrocytes with 1 µg/mL CeNPs reduced OGD/R-induced necrosis to 18%, and early and late apoptoses were detected in 69% and 11% of astrocytes, respectively (Figure 6, CeNPs 1 µg/mL + OGD/R). At the same time, no more than 2% of astrocytes remained viable (Figure 6C). An increase in the concentration of CeNPs to 10 µg/mL led to an increase in the number of viable cells up to 60% due to a decrease in astrocytes at the early and late stages of apoptosis to 24% and 6%, respectively (Figure 6—CeNPs 1 µg/mL + OGD/R). Necrosis was detected in 10% of astrocytes (Figure 6C).

Thus, cerium oxide nanoparticles are most effective in protecting cerebral cortex astrocytes from OGD/R-induced death at a concentration of 10 μg/mL, when the most pronounced increase in baseline and OGD/R-induced expression of genes encoding protective proteins occurred. Under the effect of 10 μg/mL CeNPs, there was a significant increase in the number of viable astrocytes after exposure to OGD/R, whereas CeNPs at a concentration of 1 μg/mL reduced necrotic cell death, but astrocytes were at an early stage of apoptosis.

### 2.4. The Effects of CeNPs on Basal and Ischemia/Reoxygenation-Induced Expression of Genes Encoding Selenoproteins and Selenium-Containing Proteins

**Figure 7:**
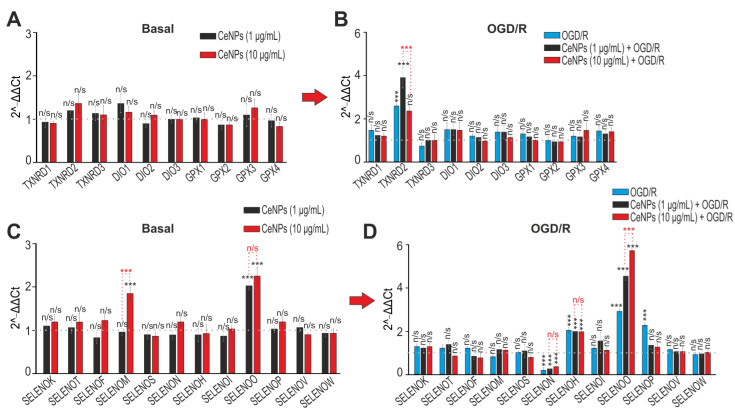
The effect of 24 h pre-incubation of cortical astrocytes with 1 or 10 μg/mL CeNPs on the basal (**A**,**C**) and OGD/R-induced (**B**,**D**) expression of genes encoding selenium-containing proteins (**A**,**C**) and selenoproteins (**B**,**D**). Gene expression in intact cells (control) is marked with a dashed line. Statistical significance was assessed using an ordinary two-way ANOVA test with Geisser–Greenhouse correction. Comparison of experimental groups vs. control: n/s—no significant differences (*p* > 0.05), *** *p* < 0.001, *n* = 3. Comparison of experimental groups with each other is indicated in red.

It is well known that selenium-containing proteins and selenoproteins are involved in the regulation of the redox status of cells and their fate under stress [32,33,34]. Moreover, we have repeatedly shown that the expression of these proteins changes significantly under OGD/R conditions, which makes a significant contribution to the cytoprotective effect of astrocytes. Astrocytes, have been shown, to be able to increase the expression of antioxidant selenoproteins during ischemia. Therefore, one of the aims of this work was to study the effect of 1 and 10 µg/mL CeNPs on the expression of mammalian selenoproteins in astrocytes, including under OGD/R conditions. We analyzed the mRNA expression patterns of 22 selenoproteins. Since the antioxidant role of CeNPs has been repeatedly proven [35,36,37,38,39], it was important to understand whether the expression pattern of a number of selenoproteins, which are key redox regulators in the cell: thioredoxin reductase and glutathione peroxidase, would change (Figure 7A–C). From the RT-PCR results shown in Figure 7A, it can be concluded that CeNPs did not cause changes in the mRNA expression levels of the four glutathione peroxidases studied. Similar results can be observed for two thioredoxin reductases: TXNRD1 and TXNRD3 (Figure 7A). However, our results demonstrate a more than two-fold increase in TXNRD2 mRNA expression under OGD conditions compared to the control (Figure 7B). At the same time, 24 h pre-incubation of astrocytes with CeNPs at a concentration of 1 µg/mL did not cause a decrease in this level of mRNA expression, but, on the contrary, to an increase. CeNPs at a concentration of 10 µg/mL brought these parameters to the values observed under OGD conditions (Figure 7B); however, the level of expression of this selenoprotein mRNA remained high compared to control (intact) cells.

It has been reliably shown that thyroid hormones play a crucial role in the development of the brain and influence it’s functioning throughout life [40,41,42,43]. Astrocytes, which are 10-fold more numerous than neurons, supply T3 to other nerve cells by deiodination of T4 [44,45]. Therefore, it was important to follow whether the application of CeNPs affects the expression of three deiodinases (DIO1, 2 and 3). However, according to our results, we did not observe any significant effect of CeNPs on the mRNA expression of these selenium-containing proteins (Figure 7B).

The classical selenoproteins are widely distributed in brain cells, are located in the cytosol, and are also found in the endoplasmic reticulum. These selenoproteins regulate numerous cell functions, including endoplasmic reticulum stress, Ca^2+^ homeostasis, apoptosis, and redox status [32,33,34]. When the expression pattern of selenoprotein P (SELENOP) in astrocytes was analyzed in relation to the concentration of CeNPs, the following regularity was observed: in the absence of OGD, CeNPs did not affect the level of expression of this selenoprotein (Figure 7C), while under OGD conditions, the expression of its mRNA increased two-fold, and pre-incubation of astrocytes with 1 and 10 µg/mL did not decrease SELENOP mRNA expression (Figure 7D). Similar results were obtained when SELENOH mRNA expression was analyzed: an almost two-fold increase in its expression levels under OGD/R conditions without and with pre-incubation for 24 h with 1 and 10 µg/mL CeNPs; however, under normal conditions, CeNPs had no effect on the expression of this selenoprotein mRNA.

In this series of experiments, it was also found that under OGD conditions, a sharp decrease in SELENOP expression in astrocytes was observed (Figure 7D); however, pre-incubation of cells with 1 and 10 µg/mL CeNPs contributed to an increase in the expression of this gene. At the same time, the application of CeNPs to intact astrocytes did not significantly affect its expression (Figure 7C). In this study, it was found that 1 and 10 µg/mL CeNPs could normally increase the expression of selenoprotein O (SELENOO) by 2 or more times (Figure 7C). Additionally, 10 µg/mL tended to increase the expression of the basic expression of SELENOM (Figure 7C). Despite the fact that the expression of this gene also increased under OGD conditions, pre-incubation of astrocytes with CeNPs did not normalize this parameter (Figure 7D).

Thus, we can make a preliminary conclusion that CeNPs in astrocytes are themselves able to enhance mRNA expression of only one of the selenoproteins analyzed in this work, namely, SELENOO. Since this information is based only on the results of RT-PCR, it is difficult to speak reliably about this regulation, and further studies are needed. However, pre-incubation of astrocytes with CeNPs, especially at high concentrations, significantly affected the expression of a number of selenoproteins (TXNRD2, SELENOH, SELENON, SELENOP) under oxygen–glucose deprivation/reoxygenation. Since all these selenoproteins are involved in the regulation of redox homeostasis in the cell, the effect of CeNPs, mediated through the regulation of the expression of these proteins, is neuroprotective by activating complex antioxidant defense mechanisms in astrocytes.

## 3. Discussion

### 3.1. Nanocerium as a Regulator of the Redox Status of Brain Cells

CeNPs have been found to be able to reduce motor symptoms in a mouse model of multiple sclerosis, a debilitating autoimmune degenerative disease of the central nervous system [46]. In vitro models have shown that CeNPs protect cortical neurons and spinal cord neurons from oxidative stress [47,48]. However, there is evidence that a single intravenous administration of CeNPs with a diameter of 5 nm had a pro-oxidant effect on brain cells 30 days after administration, while the nanoparticles did not pass the blood–brain barrier. However, 30 nm CeNPs with the same application method resulted in a so-called hierarchic oxidative stress response in the rat hippocampus with a peak at day 30 and resolution at day 90 after exposure [49]. Moreover, the induction of ROS-independent apoptosis under the effect of nanocerium was shown in human monocyte cell cultures [18,50]. Thus, the effects of nanocerium on brain cells are controversial. Probably, the cytoprotective or pro-apoptotic effect of CeNPs depends on their physical properties and method of preparation.

Thus, in our experiments, we found that CeNPs at concentrations of 30 µg/mL and higher induced ROS production in astrocytes of the cerebral cortex that was comparable to OGD-induced ROS production. Preliminary 24 h incubation of astrocytes with CeNPs concentrations up to 10 µg/mL did not cause ROS production, but led to a change in the expression profile of genes encoding antioxidant proteins, which leads to suppression of OGD-induced ROS production.

The toxic or cytoprotective effect of cerium oxide nanoparticles in astrocyte cultures can also be regulated by their need to enter the cell. The use of high doses of nanocerium causes an influx of high concentrations of CeNPs into cells, and 24 h pre-incubation of astrocytes with low doses of nanoparticles contributes to their gradual accumulation and induction of the cell preconditioning effect. It has been shown that the physiological effects of nanoparticles are determined by different membrane-bending energies during endocytosis [51]. It was shown on CHO-K1 cells that nanocerium (3 nm) accumulates mainly in the intracellular space, and the cellular uptake of nanoparticles is dose- and time-dependent. Saturation of the cell with nanocerium occurs at a concentration of approximately 0.125 mM. It was found that low doses of nanocerium (0.1 and 0.3 mg/kg^−1^) did not decrease infarct volume, whereas CeNPs at concentrations of 0.5 and 0.7 mg/kg^−1^ significantly reduced infarct volume up to 50%. However, higher doses of CeNPs (1.0 and 1.5 mg/kg^−1^) showed no protective effect against stroke [17]. The use of CeNPs in stroke should take into account the therapeutic window of application and be used in strictly optimal doses to avoid negative effects. Using BV-2 microglial cells, it was shown that CeNPs penetrate them with high efficiency after 30 min, and this process increases with increasing incubation time. In the progenitor line of C17.2 neurons at the stage of active proliferation, it was found that CeNPs are predominantly localized in membrane bound structures and to a lesser extent were found free in the cytoplasm, and in very low amounts in nuclei [22].

CeNPs can also accumulate in lysosomes and mitochondria, with an internalization coefficient of 0.65 and 0.15 for lysosomes and mitochondria, respectively [16]. Since lysosomes and mitochondria make a huge contribution to ROS generation, including during OGD/R, the accumulation of CeNPs in these organelles can make a significant contribution to protection against oxidative stress [16]. It was found that ROS production increased 5-fold after OGD/R in BV-2 microglial cells, and nanocerium suppressed OGD/R-induced ROS production by 35.7 and 50% when using 0.32 or 0.64 µg/mL CeNPs, respectively [16]. In our experiments with astrocytes, which are the most numerous types of glial cells in the brain, it was found that nanocerium at a concentration of 10 μg/mL did not cause a toxic increase in ROS production in an acute experiment, but after 24 h pre-incubation it most effectively suppressed the OGD-induced growth of ROS production.

CeNPs are known to act as catalysts that mimic a number of redox enzymes, including superoxide dismutase, catalase, peroxidase, phosphotriesterase, phosphatase, and oxidase, which can scavenge reactive oxygen species (ROS) [52,53,54]. On the other hand, it has been shown that CeNPs are able to induce strong oxidative stress in human bronchoalveolar carcinoma and human hepatocellular carcinoma [55]. CeNPs injections have been shown to increase basal expression and activity of SOD and GSH-Px in myocardial tissue, which protects cardiomyocytes from oxidative stress during ischemia/reperfusion [56]. In our experiments, pre-incubation of astrocytes with CeNPs (10 μg/mL) resulted in an increase in baseline and OGD/R-induced expression of Nrf2, Hif1α, and Cat1, which are involved in the suppression of oxidative stress. The protective effect of nanocerium in paraquat-induced brain injury was shown. The use of nanocerium led to an increase in the antioxidant defense mechanism via increasing the total thiol content and total antioxidant capacity, thereby decreasing the lipid peroxidation, oxidative DNA damage as well as caspase-3 activity. Besides, the mRNA levels of Nestin and Neurod1 has also been increased in the brain samples upon nanoceria treatment [57].

### 3.2. Regulation of mRNA Expression of Selenoproteins by Nanocerium

However, a huge number of enzymes, including selenoproteins, are involved in protection against oxidative stress. It is known that the neuroprotective effects of selenoproteins are mediated in part by complex antioxidant defense mechanisms that are critical for protection against stress-inducing ROS. Therefore, it was important to follow how the expression of selenoproteins in astrocytes changes, including under OGD conditions.

We found that CeNPs supplementation did not cause changes in the levels of mRNA expression of the four studied glutathione peroxidases, which are important cellular redox regulators that also play a key role in redox homeostasis, which is necessary for the restoration of peroxide in the intestine and blood, phospholipid peroxide. Similar results can be observed for two thioredoxin reductases: TXNRD1 and TXNRD3, except for TXNRD2, whose expression increased more than two-fold under OGD conditions. It is known that TXNRD2 reduces oxidized thioredoxin, acting mainly in mitochondria [32,33,58]. It has been shown that TXNRD2-deficient mice exhibited anterior neural tube defects and embryonic lethality, while overexpression of this enzyme, along with TXNRD1, promoted neuronal survival after various stressful events [34,59]. Increased expression of TXNRD2 mRNA under OGD conditions, even with pretreatment of astrocytes with CeNPs at the concentrations studied, most likely had an antioxidant effect and was aimed at the survival of astrocytes under stressful conditions.

It is well known that SELENOP has strong antioxidant properties, which has been demonstrated in a number of studies, for example, in rat prostate cells [60], human endothelial cells [61], and astrocytes [62]. In our experiments under OGD conditions, increased expression of its mRNA also had an antioxidant effect; however, pre-incubation of astrocytes with CeNPs, which also have antioxidant properties, reduced the level of SELENOP expression. Our results are consistent with data on rat pheochromocytoma cells (PC12 cell line), which are a valuable model for neuronal differentiation [63].

We obtained similar results for the selenoprotein SELENOH, which is a novel nucleolar oxidoreductase and has the redox property of DNA binding [64]. It was also shown that SELENOH is able to increase the level of glutathione, glutathione peroxidase, and total antioxidant activity in hippocampal neurons of HT22 mice [65]. The authors found that SELENOH significantly slows down the collapse of the mitochondrial cytoskeleton and restores the structural integrity of damaged mitochondrial components. In addition, this selenoprotein counteracted glutamate toxicity in HT22 neurons similarly to the protection afforded by elemental selenium [66]. It is possible that the increased expression of SELENOH mRNA caused by oxygen–glucose deprivation is aimed at enhancing the activity of antioxidant enzymes in astrocytes. At the same time, CeNPs themselves did not significantly affect the change in the mRNA expression of this selenoprotein.

We also showed that CeNPs affected SELENON mRNA expression, enhancing it under OGD conditions. It is well known that this selenoprotein is a type II glycoprotein that localizes in the ER and determines the level of calcium ions in SERCA through a redox-mediated mechanism. In muscle, a decrease in SELENON activity changes calcium homeostasis in the ER and triggers ER stress [67,68]. It is possible that pre-incubation of astrocytes in CeNPs protects cells from hyperoxidation and disturbance of calcium homeostasis through a redox mechanism.

We have also shown for the first time that CeNPs are able to enhance the expression of SELENOO-selenoproten, which is involved in a previously undocumented mechanism of redox regulation, by which this protein protects cells from oxidative damage. SELENOO, along with HYPE (protein adenylyltransferase), catalyzes the binding of ATP and the transfer of AMP from ATP to serine, theronine, and tyrosine residues in protein substrates, followed by post-translational modification called AMPylation [69]. Similar to TXNRD2 and GPX4, SELENOO is localized in mitochondria and contains a signal peptide at the N-terminus followed by a protein kinase-like domain. However, this selenoprotein was shown to be a pseudokinase, because it lacks the catalytic aspartate, which serves as a common base for deprotonation of the hydroxyl group of the substrate [70]. Given that AMPylation is a still unknown lysosomal post-translational modification that plays an increasingly important role in the development of the nervous system, it is difficult to assert that this process is regulated by cerium oxide nanoparticles through the activation of SELENOO in astrocytes. However, the data obtained can serve as an important prerequisite for the study of the molecular mechanisms involved in this post-translational modification, with the active participation of CeNPs.

### 3.3. Interrelation of Nanocerium with Calcium Signaling of Astrocytes

The protective effect of nanocerium (20 nm in diameter) on endothelial cells under H_2_O_2_-induced oxidative stress was shown by the penetration of the nanoparticles into cells via caveolae- and clathrin-mediated endocytosis was shown [71]. Similarly, for selenium nanoparticles with a diameter of 100 nm, we found two pathways of their penetration into cells (glioblastomas and astrocytes) using micropinocytosis and clathrin-associated endocytosis, which resulted in the generation of Ca^2+^ signals in an acute experiment [27,72].

The cerium oxide nanoparticles that we used in this experiment were 100 nm in diameter, and their application also led to the generation of Ca^2+^ signals by astrocytes, probably due to rapid endocytosis into the cells, which was shown for the first time. However, the close relationship between the Ca^2+^ dynamics of brain cells and their death has long been known, including several works showing the modulatory role of nanoselenium on the Ca^2+^ signaling system of brain cells. Spherical CeNPs reduce neuronal death and cytosolic calcium dysregulation by maintaining the antioxidant system in mild traumatic brain injury [5].

It was shown that pretreatment of brain cells with 10 nM CeNPs reduced mitochondrial fragmentation induced by beta-amyloid, endogenous peroxynitrite and neuronal cell death, promoted the formation of astrocyte monolayer and neuronal outgrowth. Simultaneously, there was an increase in the level of catalase and a decrease in the level of glutathione [2]. Ischemia is associated with the release of a large amount of glutamate into the extracellular space. Neurons are most vulnerable to glutamate excitotoxicity, while astrocytes are more resistant [73]. There is evidence that CeNPs are able to regulate the dynamics of [Ca^2+^]_i_. Uninjured neurons stimulated with 100 µM glutamate responded with an average change in [Ca^2+^]_i_ of 203 ± 12 nM, which was similar in uninjured neurons treated with CeNPs. Twenty-four and 48 h after mild injury, the response to glutamate was decreased, indicating impaired signal transduction [74]. However, in cells treated with CeNPs 1 h after mild injury, the response to glutamate was the same as in uninjured cells. Twenty-four hours after moderate injury, the neuronal response to glutamate was dramatically increased, indicating excitotoxicity. Then, at 48 h after injury, the response to glutamate was depressed, below normal levels, consistent with our previous reports. In contrast, cells treated with CeNPs showed no aberrations in glutamate signaling 24 and 48 h after mild or moderate in vitro injury. These results indicate that CeNPs, administered 1 h after injury, can block injury-induced abnormal glutamate signaling, possibly preserving neuronal function after traumatic injury [5]. At the same time, there are no data on the effect of CeNPs on the Ca^2+^ dynamics in astrocytes.

We have shown that CeNPs in the concentration range of 3–10 µg/mL administered 24 h before OGD induction prevented a global increase in [Ca^2+^]_i_ in the astrocyte, which correlated with the suppression of necrosis. Interestingly, lower or higher concentrations of CeNPs had no positive effects on OGD-induced Ca^2+^ dynamics and astrocyte death, which is in good agreement with our results on the dose-dependent effects of nanocerium on basal and OGD-induced ROS production.

It is known that the incubation of brain cells with antioxidants of both protein origin [74] and non-protein origin alone or as part of nanocomplexes [28,29] regulates the level of not only the redox status of cells, but also the levels of pro-apoptotic and pro-inflammatory proteins. Nanocerium after 24 h of exposure affected the baseline or OGD/R-induced expression of only one protective protein, IL-10, associated with the suppression of inflammation. However, necrosis and apoptosis decreased, probably due to the effects of CeNPs on the redox status of the cell and suppression of the OGD-induced increase in [Ca^2+^]_i_.

Ca^2+^ ions are known to regulate the activity of hundreds of intracellular proteins [75,76]. An increase in [Ca^2+^]_i_ can have both acute effects (in our experiments, OGD-induced global increase in [Ca^2+^]_i_ and death of astrocytes) and delayed effects aimed at activation of protective signaling cascades. The delayed effects of the [Ca^2+^]_i_ increase are associated with changes in the expression of genes and their encoding proteins [77,78]. Sources of [Ca^2+^]_i_ increase in cells can be the mobilization of Ca^2+^ ions from intracellular depots (primarily the endoplasmic reticulum or acid pools) or Ca^2+^ entry from outside through the channels of the plasma membrane [79,80]. In addition, the Ca^2+^ ions entry from the outside can function to maintain the amplitude of the Ca^2+^ signal even if the trigger was the mobilization of Ca^2+^ from intracellular pools [81]. In the case of nanocerium, it can be assumed that the signaling cascade is activated in analogy with selenium nanoparticles, when the endocytosis of nanoparticles activates the phospholipase C signaling cascade and leads to the release of Ca^2+^ ions from the endoplasmic reticulum [25]. At the same time, nuances in signaling under the action of nanocerium are possible, and the mechanisms of activation of the Ca^2+^ signaling system require further experiments.

### 3.4. Comparative Analysis of Cerium Nanoparticles and Selenium Nanoparticles under the Action on Cortical Astrocytes. Limitations of Nanocerium

Cerium and especially nanocerium is not a vital element. Nanocerium is used as a drug carrier and, as shown by numerous works, is not toxic to healthy cells and tissues [82]. Nanocerium does not enter metabolism and is excreted from the body. However, as our studies have shown, nanocerium is capable of exerting a prooxidant effect on cortical astrocytes at concentrations of 30 µg/mL and above. At the same time, CeNPs at concentrations of 10–30 µg/mL are able to protect astrocytes from oxidative stress under oxygen–glucose deprivation; there is a very “narrow” therapeutic window between the positive and negative effects of nanocerium on brain cells during ischemia. At the same time, our previous studies have shown that selenium nanoparticles (SeNPs), also obtained by laser ablation, are able to protect cortical astrocytes from oxidative stress under OGD or the effect of exogenous hydroperoxide in the concentration range of 0.5–10 µg/mL [29]. A distinctive feature of SeNPs is the fact that selenium is a vital natural trace element that enters into metabolism [83,84]. Nanocerium, similar to nanoselenium, activates the Ca^2+^ signaling system of cortical astrocytes, but for CeNPs the EC50 value is 20 ± 0.2 µg/mL, while SeNPs induce Ca^2+^ signals in astrocytes at EC50 = 0.58 µg/mL [27]. Of course, SeNPs exert their anti-apoptotic and antioxidant effects by increasing the expression of selenium-containing proteins and selenoproteins [28], while nanocerium affects the expression of only three proteins. Nevertheless, despite the obvious advantages of selenium nanoparticles, there is still a need to study the positive and negative aspects of the action of nanoparticles of various origins. It is of interest to investigate the stability and time of excretion from the body of both types of nanoparticles, which will require studies at the in vivo level.

## 4. Materials and Methods

Experimental protocols were approved by the Bioethics Committee of the Institute of Cell Biophysics. Experiments were carried out according to Act708n (23 August 2010) of the Russian Federation National Ministry of Public Health, which states the rules of laboratory practice for the care and use of laboratory animals, and the Council Directive 2010/63 EU of the European Parliament on the protection of animals used for scientific purposes.

### 4.1. Preparation and Characterization of Cerium Oxide Nanoparticles

**Figure 8:**
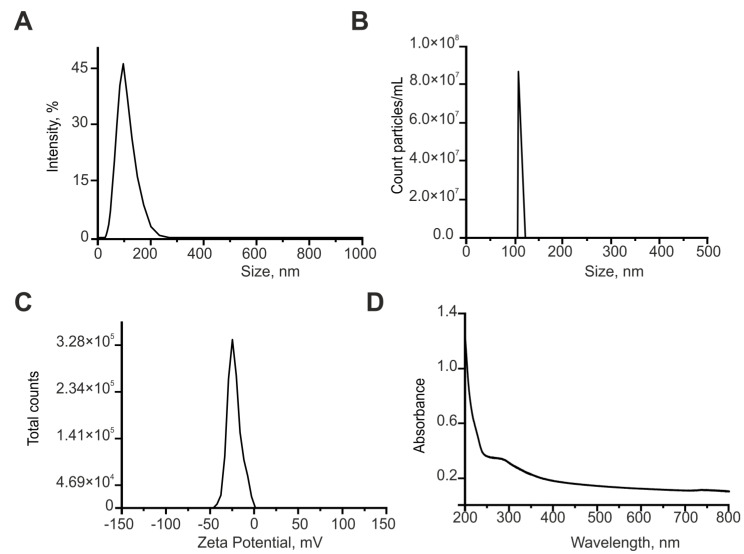
Characterization of cerium oxide nanoparticles. (**A**,**B**)—The nanoparticles size (**A**) and concentration (**B**). (**C**)—The zeta potential profile of the nanoparticles. (**D**)—Optical absorption spectrum of an aqueous colloid of cerium oxide nanoparticles.

Cerium oxide (CeO_2_) nanoparticles were obtained by laser ablation in MQ water. A solid target was placed on the bottom of the cuvette under a thin layer of water. In this state, the solid target was irradiated with laser radiation (λ = 1064 nm, T = 4–200 ns, f = 20 kHz, *p* = 20 W, Ep = 1 mJ). The laser beam was targeted to the target using a galvanomechanical scanner. Depending on the characteristics of laser radiation, the speed and trajectory of the laser beam; it is possible to obtain colloidal solutions of cerium oxide nanoparticles with specified geometric parameters [85]. Nanoparticle concentration and hydrodynamic radius were evaluated using Zetasizer Ultra Red Label (Malvern, UK). The morphology of nanoparticles was studied using a 200FE transmission electron microscope (Carl Zeiss, Jena, Germany). The optical properties of the dispersions were studied using a GBC Cintra 2020 UV-Vis spectrometer (UVISON Technologies Limited, London, UK) in the wavelength range of 200–900 nm [86]. The resulting cerium oxide nanoparticles have a monomodal size distribution. The average nanoparticle size is approximately 108 nm and the half-width is in the range of 92–125 nm (Figure 8A). The concentration of nanoparticles in the colloid is 10^7^ NPs/mL (Figure 8B). The zeta potential profile of the nanoparticles was also determined, peaking at −24.64 mV (Figure 8C). The composition of the resulting nanoparticles corresponding to cerium oxide was confirmed by absorption spectroscopy (Figure 8D). Since Ce is a chemically active metal (the electrochemical potential of Ce is ϕ0 = −2.336), its particles are rapidly oxidized in water. In the optical spectrum of the obtained colloid, a strong scattering is observed in the entire range with an increase in the UV region and an absorption peak with a maximum at a wavelength of 280 nm.

**Figure 9:**
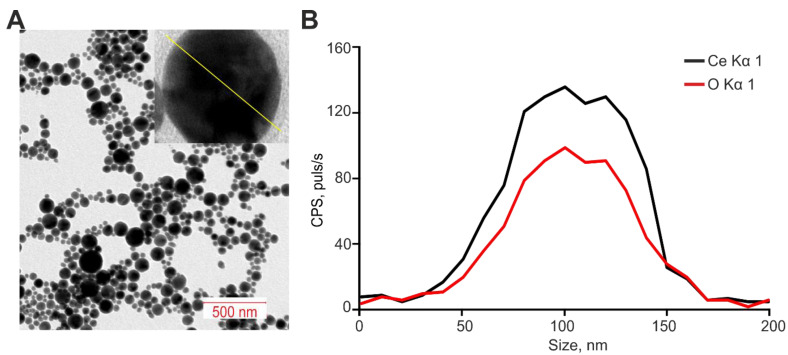
Characterization of cerium oxide nanoparticles. (**A**)—TEM microphotograph of cerium oxide nanoparticles. Above—TEM image of cerium oxide nanoparticles, analysis section is indicated by line. (**B**)—Elemental analysis of cerium oxide nanoparticles. Nanorods profile by Ce Kα1 and O Kα1.

The morphology of cerium oxide nanoparticles was studied using transmission electron microscopy. It is shown that the nanoparticles have a spherical shape (Figure 9A). Separately, elemental analysis was also performed (Figure 9B). The chemical composition of the nanorods has been confirmed.

### 4.2. Primary Cortical Culture

Astroglial cell cultures were isolated from the brains of 1–2-day-old mouse according to the modified McCarthy and de Vellis protocol [87]. The brains were extracted, the cerebral cortex was separated, the meninges were removed, and the tissue was minced and incubated in 0.05% trypsin-EDTA solution at 37 °C for 30 min. After enzymatic digestion, the tissues were washed twice in PBS and then dissociated by glass Pasteur pipette in a culture medium consisting of DMEM (PanEco, Moscow, Russia), 1 g/L D-glucose, and 10% FBS (Biosera, Kansas City, MO, USA), with the addition of 2 mM glutamine (PanEco, Russia). The suspension of cells was transferred on ventilated culture vials (Costar, Washington, DC, USA) precoated with poly-D-lysine (10 μg/mL). The cells were cultivated at 37 °C and 5% CO_2_. After 5–6 days, the cultures were shaken on an orbital shaker at 200 rpm for 16 h to detach and remove microglia. After 10 to 20 days, in vitro astrocytes were used for experiments.

Neuroglial cortical cultures were prepared in a similar manner as astroglial cultures, but cells were cultured in Neurobasal-A medium containing glutamine, B-27 (2%, Thermo Fisher Scientific, Waltham, MA, USA, RRID: CVCL_A315) and gentamicin (20 μg/mL, Sigma-Aldrich, St. Louis, MI, USA, Cat #G1397) [25].

### 4.3. Fluorescent Ca^2+^ Measurements

To detect the changes in [Ca^2+^]_i_, cell cultures were loaded with Fura-2 (4 µM; 40 min incubation; 37 °C). The cells were stained with the probe dissolved in Hank’s balanced salt solution (HBSS) composed of (mM): 156 NaCl, 3 KCl, 2 MgSO_4_, 1.25 KH_2_PO_4_, 2 CaCl_2_, 10 glucose, and 10 HEPES, pH 7.4. To measure [Ca^2+^]_i_, we used the system based on an inverted motorized microscope Leica DMI6000B (Leica, Wetzlar, Germany) with a high-speed monochrome CCD-camera HAMAMATSU C9100. For excitation and registration of Fura-2 fluorescence, we used FU-2 filter set with excitation filters BP340/30 and BP387/15, beam splitter FT-410, and emission filter BP510/84. Illuminator Leica EL6000 with a high-pressure mercury lamp was used as a source of excitation light. All the Ca^2+^ signals are presented as a 340/380 ratio of Fura-2 fluorescence.

### 4.4. Measurement of ROS Production

For simultaneous recordings of ROS production, cortical astrocytes were loaded with H2DCF-DA (10 µM, 20 min incubation; 37 °C). In addition, in order to evaluate the intensity of mitochondrial staining of the ROS indicators in the performed experiments, the double staining of cell cultures with H2DCF-DA/MitoTracker Red FM was performed. The final concentration of MitoTracker Red FM was 200 nM (20 min incubation; 37 °C). After incubation with the dyes, cells were washed three times before the experiment. To measure the ROS generation, the system based on the inverted motorized microscope Leica DMI6000B (Leica, Wetzlar, Germany) with a high-speed monochrome CCD-camera HAMAMATSU C9100 and a high-speed light filter replacing system Leica’s Ultra-Fast Filter Wheels with a replacing time 30 s was used. For the excitation of DCFH2-DA, an L5 filter set with the excitation filter BP480/40, dichroic mirror 505, and emission filter 527/30 was used. In order to prevent nonspecific photo-oxidation of the ROS indicators, laser power was decreased to 3%–5%. The shape and speed of ROS production rates under oxygen–glucose deprivation (OGD) or application of CeNPs were determined.

To evaluate H2DCF-DA/MitoTracker Red colocalization, an inverted confocal microscope (Leica TCS SP5, Wetzlar, Germany) was used. For the excitation of DCFH2-DA, an argon laser with line 488 nm was used. Emission was collected in the range of 510–590 nm. MitoTracker Red FM is a far-red-fluorescent dye; therefore, a 633 nm He–Ne laser for its excitation was used. Emission was collected in the range of 655–700 nm.

To register the total production of ROS in cells and to reveal the concentration effects of CeNPs the cortical astrocytes were grown in 24-well plates for 3 days. Next, various concentrations of the CeNPs were added to the culture medium for 24 h, after that, the cells were washed and loaded with H2DCF-DA (20 µM, 30 min incubation; 37 °C). The cells were then washed with HBSS, and baseline DCF fluorescence was recorded using an automated multiplate reader (Spark™ 10M multimode microplate reader; Tecan Trading AG, Zurich, Switzerland, with the SparkControl 3.2 software). After detecting the base level of the fluorescence intensity, the glucose-free medium was replaced with the addition of the oxygen scavenger sodium dithionite to initiate the ROS production. Recording was performed over 40–50 min. To avoid photodestruction of the probe and to avoid the photodynamic production of ROS, DCF fluorescence was recorded once every 5 min. The ImageJ, Origin 8.5, and Prism GraphPad software 8.0.1 (GraphPad Software, San Diego, CA, USA, RRID: SCR_002798, V 244 were used in order to analyze data, create graphs, and perform statistical tests. All values are given as the mean ± SEM. All presented data were obtained from at least three cover slips and two to three different cell preparations.

### 4.5. The Technique for Simulation of Oxygen–Glucose Deprivation

Oxygen–glucose deprivation (ischemia-like conditions, OGD) were obtained by omitting glucose (HBSS medium without glucose) and by displacement of dissolved oxygen with argon in the leak-proof system [25]. The level of oxygen in the medium was measured using a Clark electrode. Oxygen tensions reached values 30–40 mm Hg or less within 20 min after the beginning of displacement. OGD lasting for 40 min or 2 h were created using supplying the oxygen–glucose deprivation (OGD)-medium into the chamber with cultured cortical cells. Constant argon feed into the experimental chamber was used to prevent the contact of the OGD-medium with the atmospheric air. For experiments on registration of ROS production, OGD conditions were simulated by adding the oxygen scavenger sodium dithionite to glucose-free HBSS, and then this medium was added to the cells.

### 4.6. Assessment of Cell Viability

Propidium iodide (1 µM) was used to evaluate the number of dead cells in the cell cultures before and after OGD. The cells were stained for 5 min with the probes diluted in HBSS and then rinsed with HBSS. Fluorescence of the probes was detected with an inverted fluorescent microscope Zeiss Axio Observer Z1 using Filter Set 20. Cell death induced by OGD was assessed by propidium iodide staining (PI, 1 µM) before and after the exposures in the same microscopic field. Furthermore, we used the Ca^2+^ signals (presence or absence of a global increase in [Ca^2+^]_i_ during OGD) as an additional indicator of cell viability [25].

Hoechst 33342 (2 µM) and propidium iodide (1 µM) were used to evaluate the number of dead cells in the cell cultures before and after 2 h OGD and 24 h reoxygenation (OGD/R conditions). The cells were stained for 5 min with the probes diluted in HBSS and then rinsed with HBSS. Fluorescence of the probes was detected with an inverted fluorescent microscope Zeiss Axio Observer Z1 using Filter Set 01 and Filter Set 20. Discrimination of early and late apoptotic cells was performed according to the previously described method [88]. Cultured astrocytes were defined as apoptotic if the intensity of Hoechst 33342 fluorescence was 3–4-fold higher compared to Hoechst 33342 fluorescence in healthy cells, indicating chromatin condensation, which can occur as a result of apoptosis induction. The differences between the early and late stages of apoptosis were determined by the intensity of Hoechst 33342 fluorescence, and at the later stages of apoptosis, cells begin to show insignificant membrane permeability for PI. Five different areas of each cell culture were analyzed. Each experimental group consisted of three cell cultures from different passages.

To simultaneously monitor apoptotic and healthy cells with fluorescence microscope, an Apoptosis/Necrosis Detection Kit (ab176750, Abcam) was used. Cells were washed 1–2 times and resuspended with Assay Buffer. To detect apoptotic cells, Apopxin Green Indicator was used. Apoptotic cells were visualized using the FITC channel (Ex/Em = 490/525 nm). For staining necrotic cells, we used 7-aminoactinomycin D (Ex/Em = 550/650 nm). To detect healthy cells, CytoCalcein 450 was used and cells were visualized using the violet channel (Ex/Em = 405/450 nm).

### 4.7. Extraction of RNA

Mag Jet RNA Kit (Thermo Fisher Scientific, Waltham, MA, USA) was used for the extraction of total RNA. The RNA quality was estimated by electrophoresis in the presence of 1 μg/mL ethidium bromide (2% agarose gel in Tris/Borate/EDTA buffer). The concentration of the extracted RNA was determined with NanoDrop 1000c spectrophotometer. RevertAid H Minus First Strand cDNA Synthesis Kit (Thermo Fisher Scientific, Waltham, MA, USA) was used for reverse transcription of total RNA.

### 4.8. Real-Time Polymerase Chain Reaction (RT-qPCR)

Each PCR was performed in a 25 μL mixture composed of 5 μL of qPCRmix-HS SYBR (Evrogen, Moscow, Russia), 1 μL (0.2 μM) of the primer solution, 17 μL water (RNase-free), 1 μL cDNA. Dtlite Real-Time PCR System (DNA-technology, Moscow, Russia) was used for amplification. Amplification process consisted of the initial 5 min denaturation at 95 °C, 40 cycles of 30 s denaturation at 95 °C, 20 s annealing at 60–62 °C, and 20 s extension step at 72 °C. The final extension was performed for 10 min at 72 °C. All the sequences were designed with FAST PCR 5.4 and NCBI Primer-BLAST software (https://www.ncbi.nlm.nih.gov/tools/primer-blast/primertool.cgi, accessed on 6 July 2023). The data were analyzed with Dtlite software (DNA-technology, Moscow, Russia, https://dna-technology.com/sites/default/files/dtprime_dtlite_v06_part_2.pdf, accessed on 6 July 2023). The expression of the studied genes was normalized to gene encoding Glyceraldehyde 3-phosphate dehydrogenase (GAPDH). Data were analyzed using Livak’s method.

### 4.9. Statistical Analysis

All presented data were obtained from at least three cell cultures. All values are given as the mean ± standard error (SEM) or as individual cellular signals in experiments. Statistical analyses were performed by an ordinary two-way ANOVA test with Geisser–Greenhouse correction or paired *t*-test. Differences are significant * *p* < 0.05, ** *p* < 0.01, and *** *p* < 0.001. n/s—data not significant (*p* > 0.05). MS Excel, ImageJ, Origin 2016 (OriginLab, Northampton, MA, USA), and Prism GraphPad 8.0.1 (GraphPad Software, San Diego, CA, USA, RRID: SCR_002798, V 244) software was used for data and statistical analysis.

## 5. Conclusions

**Figure 10:**
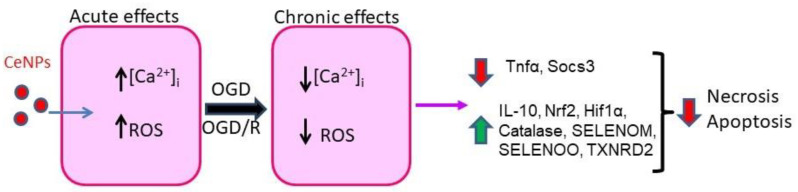
The acute (CeNPs application to astrocytes in an acute experiment) and chronic (pre-incubation of astrocytes with CeNPs for 24 h) effects of cerium oxide nanoparticles on cortical astrocytes. Red arrows show suppression of gene expression and processes of OGD/R-induced necrosis and apoptosis. Green arrows show an increase in the gene expression.

The effect of application of cerium oxide nanoparticles to cortical astrocytes in an acute experiment (acute effects) and pre-incubation with nanocerium for 24 h (chronic effects) is shown in Figure 10. CeNPs at concentrations up to 10 μg/mL were found to cause no ROS production by astrocytes, while 10 μg/mL causes a moderate increase in ROS production, which does not lead to damage and death of astrocytes in acute experiments, whereas 30 μg/mL CeNPs and above induce a strong production of ROS, comparable to the effect of ischemia on cells. Pre-incubation (Figure 10—Chronic effects) of astrocytes with CeNPs from 10 μg/mL protected them from the OGD-induced increase in ROS production due to increased expression of the Nrf2, Hif1α, and Cat genes encoding proteins that are involved in the activation of the hypoxic/ischemic preconditioning phenomenon. CeNPs were able to increase [Ca^2+^]_i_ in a dose-dependent manner with EC_50_ = 20 ± 0.2 µg/mL, probably leading to a change in the expression pattern of protective proteins and resulting in suppression of apoptosis and protection of astrocytes from ischemia/reoxygenation. In this work, for the first time, an analysis of the expression of all known selenoproteins under the action of nanocerium was carried out and key proteins, whose levels are regulated by these nanoparticles, were identified.

## Data Availability

The data presented in this study are available on request from the corresponding author.

## Supplementary Materials

The following supporting information can be downloaded at: https://www.mdpi.com/article/10.3390/ijms241814305/s1.
