# Supplementary material for: Cerium Oxide Nanoparticles Protect Cortical Astrocytes from Oxygen–Glucose Deprivation through Activation of the Ca2+ Signaling System
Source: Int J Mol Sci. 2023 Sep 19;24(18):14305. doi: 10.3390/ijms241814305 (PMC10531718; doi:10.3390/ijms241814305)
Supplement: Supplementary file 1 [file ijms-24-14305-s001.zip › ijms-2567659-supplementary.pdf]

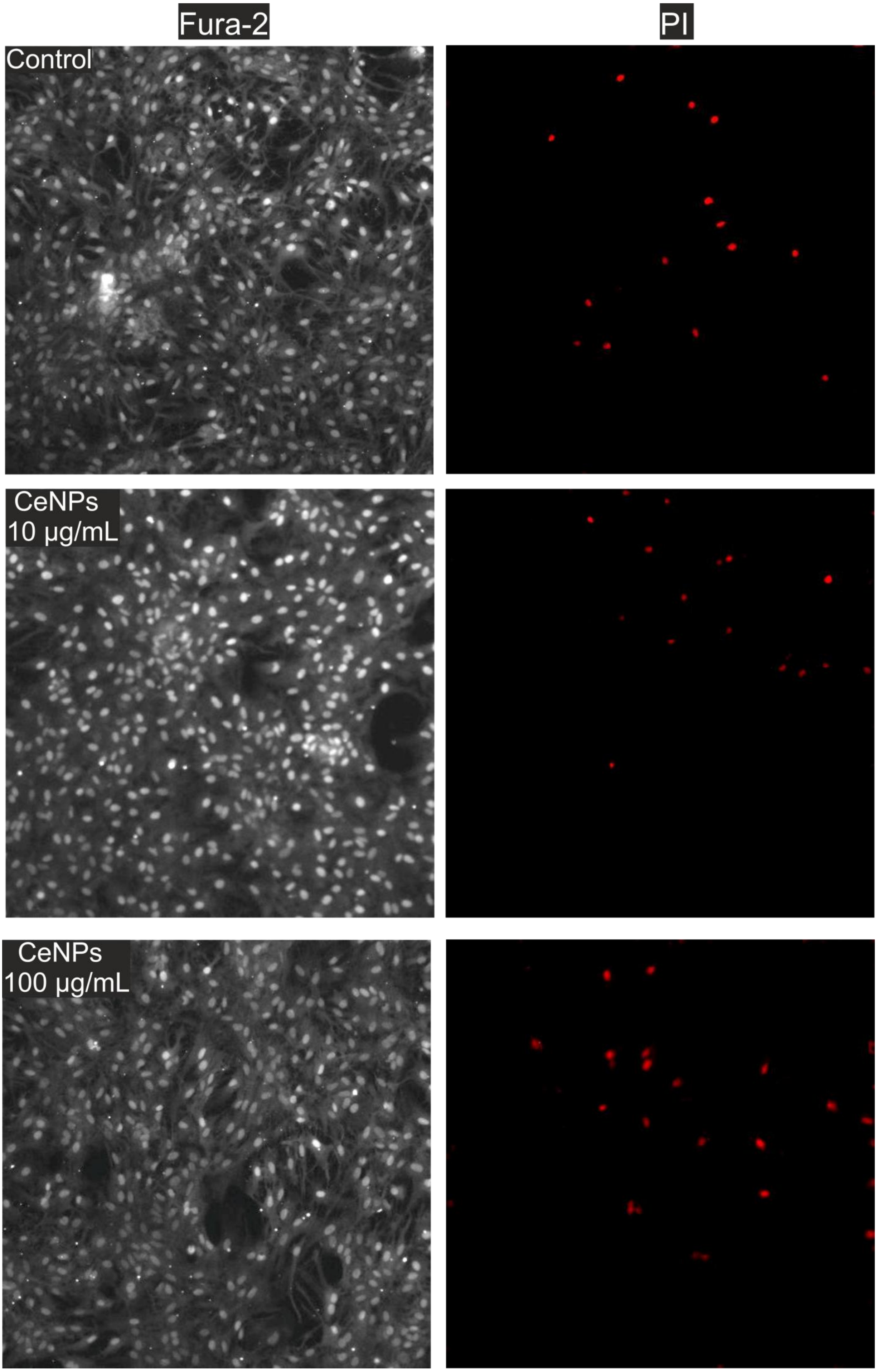
Fura-2
PI
Control
CeNPs
10 μg/mL
CeNPs
100 μg/mL

Figure S1. Application of 10 or 100 μg/mL CeNPs to cortical astrocytes in an acute experiment (time of $[Ca^{2+}]_i$ dynamics registration = 15 ± 7 min) does not cause cell death. Images of cortical cell cultures stained with the Ca^{2+}-sensitive probe Fura-2 and Propidium iodide (PI) (reflecting necrosis-type cell death) are presented. Control —astrocytes were not exposed to CeNPs. CeNPs 10 μg/mL and 100 μg/mL – application of CeNPs in an acute experiment and subsequent staining with PI. The presence of a red pseudo-color (PI fluorescence) indicates dead cells.

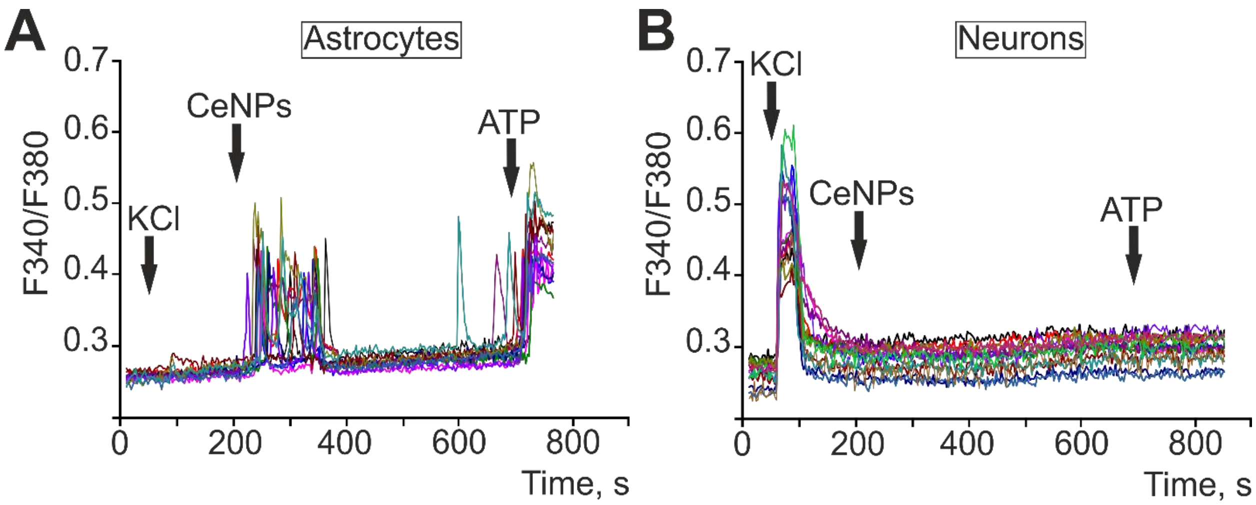

Figure S2. Upon application of 30 μg/mL CeNPs to a neuroglial culture of the cerebral cortex, astrocytes (**A**) but not neurons (**B**) respond with an increase in $[Ca^{2+}]_i$. To identify neurons at the beginning of the experiment, a short-term (30 s) application of 30 mM KCl was performed. At the end of the experiment, 10 μm ATP was added to the cells to detect astrocytes. The Ca^{2+} signals of neurons and astrocytes are shown in one experiment.

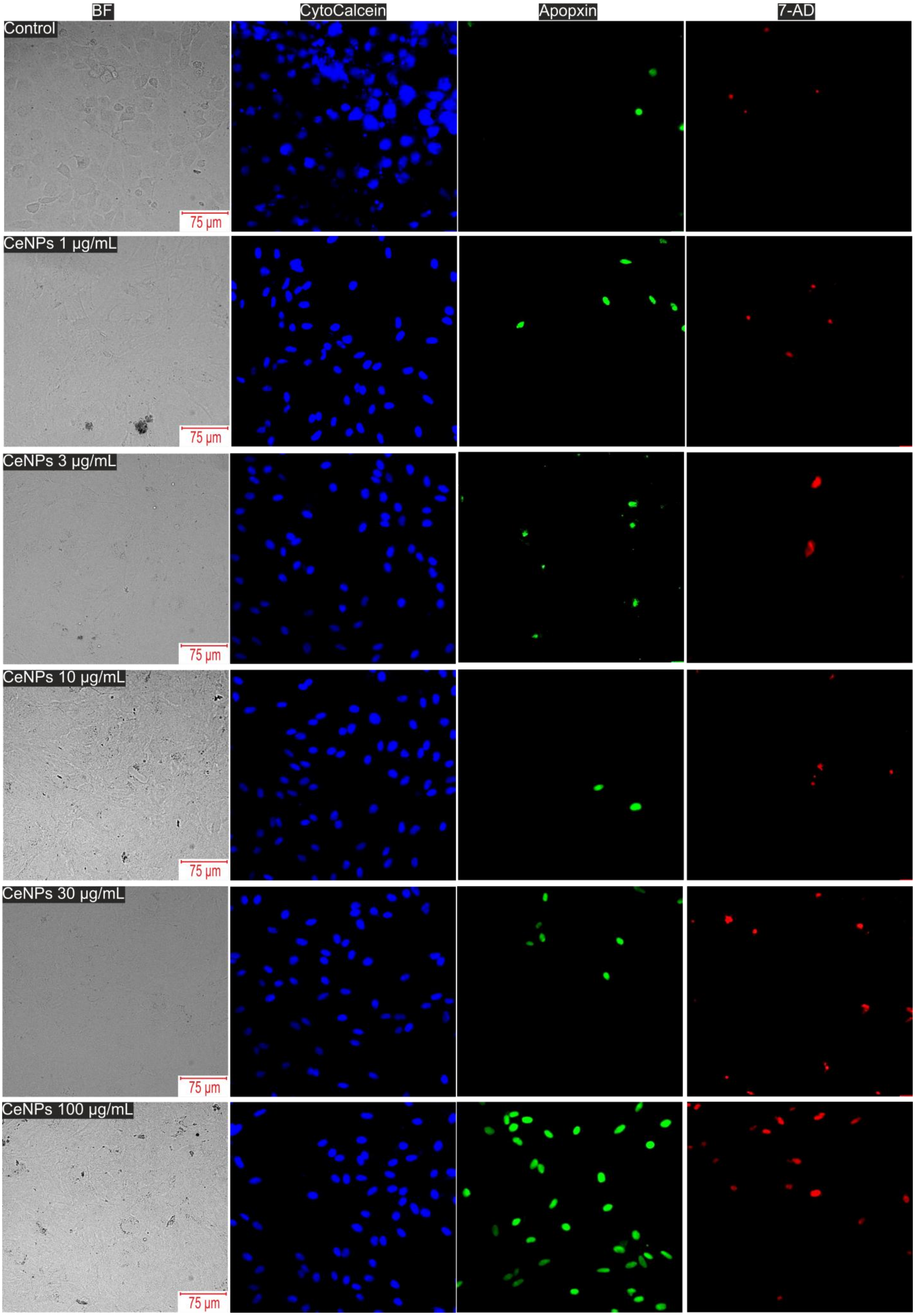

Figure S3. Effect of 24-hour incubation of cortical astrocytes with 1, 3, 10, 30 and 100 µg/mL of CeNPs on the induction of necrosis and apoptosis. Cells

staining using the Apoptosis/Necrosis Detection Kit assay. BF – bright-field microscopy, CytoCalcein – healthy cells indicator, Apopxin – apoptotic cells indicator and 7-AD (7-aminoactinomycin D) – necrotic cells indicator.
